# Supplementary material for: The interactome of intact mitochondria by cross-linking mass spectrometry provides evidence for coexisting respiratory supercomplexes
Source: Mol Cell Proteomics. 2017 Dec 8;17(2):216–32. doi: 10.1074/mcp.RA117.000470 (PMC5795388; doi:10.1074/mcp.RA117.000470)
Supplement: Supplemental Data [file supp_RA117.000470_133922_0_supp_23978_fzffwf.zip › spectra_annotation/mito_DR_spectra_annotation/465-1-6-1-3-1.pdf]

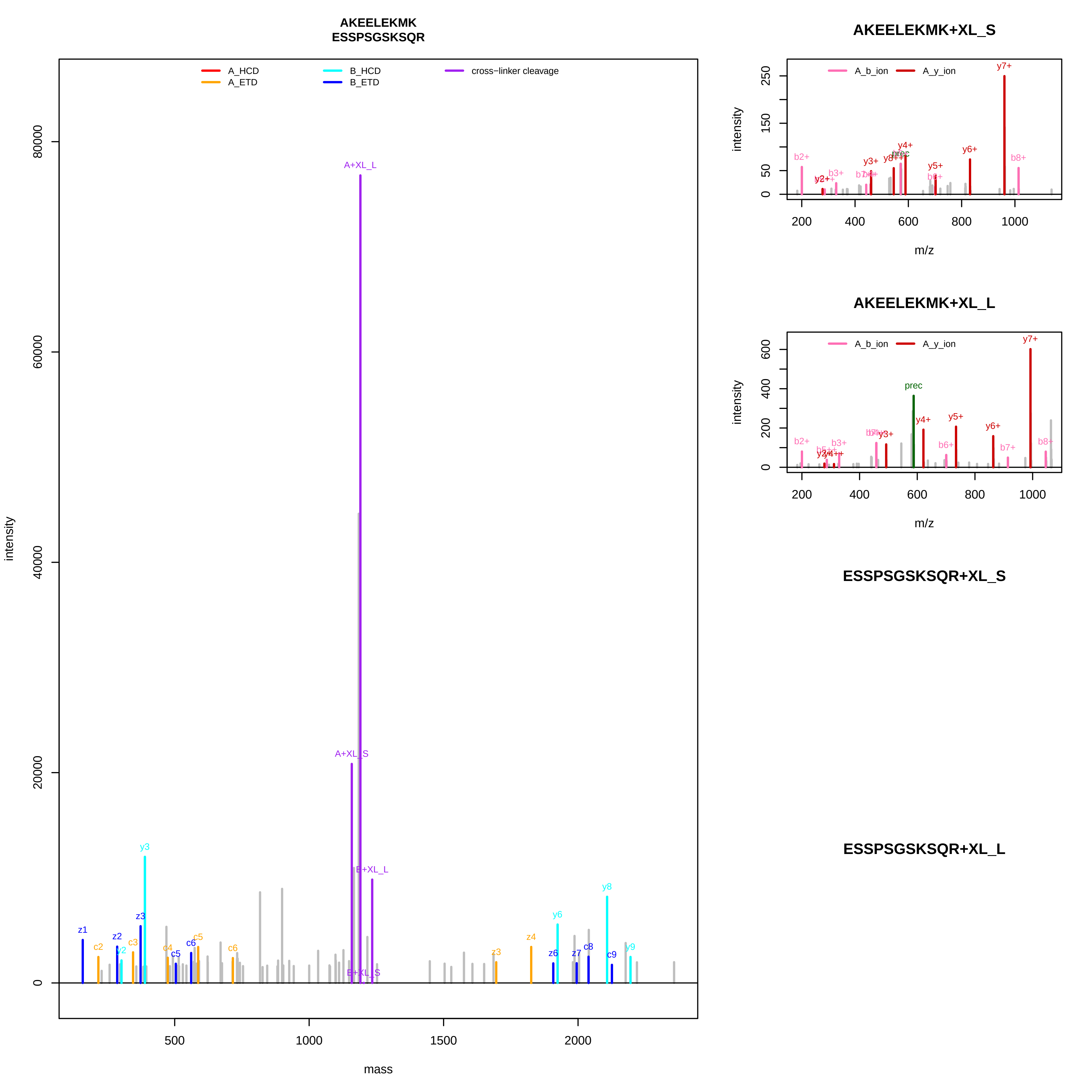
AKEELEKMK
ESSPSGSKSQR
A_HCD
A_ETD
B_HCD
B_ETD
cross−linker cleavage
intensity
mass
0
20000
40000
60000
80000
500
1000
1500
2000
A+XL_L
A+XL_S
B+XL_L
B+XL_S
z1
z2
z3
y2
y3
c2
c3
c4
c5
c6
z3
z4
z6
z7
c8
c9
y6
y8
y9
AKEELEKMK+XL_S
A_b_ion
A_y_ion
m/z
prec
y7+
AKEELEKMK+XL_L
ESSPSGSKSQR+XL_S
ESSPSGSKSQR+XL_L
